# Specific Collagen Peptides in Combination with Resistance Training Improve Body Composition and Regional Muscle Strength in Premenopausal Women: A Randomized Controlled Trial

**DOI:** 10.3390/nu11040892

**Published:** 2019-04-20

**Authors:** Patrick Jendricke, Christoph Centner, Denise Zdzieblik, Albert Gollhofer, Daniel König

**Affiliations:** Department for Nutrition, Institute for Sports and Sports Science, University of Freiburg, Schwarzwaldstraße 175, 79117 Freiburg, Germany; christoph.centner@sport.uni-freiburg.de (C.C.); denise.zdzieblik@sport.uni-freiburg.de (D.Z.); ag@sport.uni-freiburg.de (A.G.); daniel.koenig@sport.uni-freiburg.de (D.K.)

**Keywords:** body composition, hand-grip strength, leg press, premenopausal, resistance training, collagen peptides, protein supplementation

## Abstract

The aim was to investigate the effects of resistance exercise combined with supplementation of specific collagen peptides (SCP) on body composition and muscle strength in premenopausal women. In a double-blind, placebo-controlled, randomized trial 77 premenopausal women completed a 12-week resistance training (3 day/week) and ingested 15 g of SCP or placebo on a daily basis. Changes in body composition were determined by bioelectrical impedance analysis (BIA) and muscular strength by isometric strength testing. The treatment group (TG) significantly increased (*p* < 0.001) their percentage of fat-free mass. Although the control group (CG) also showed a significant (*p* < 0.01) gain in fat-free mass from pre- to post-training, the increase in the TG was significantly higher in an RMANOVA analysis (*p* < 0.05). Regarding the change in percentage body fat, a significant decline was observed in both TG (*p* < 0.001) and CG (*p* < 0.01), with a significantly higher reduction in the TG (*p* < 0.05). Subjects receiving 15 g of collagen peptides daily also showed a significantly higher gain in hand-grip strength compared to those performing resistance training only (*p* < 0.05). In both groups, the gain in leg strength (TG = *p* < 0.001; CG = *p* < 0.01) was significant after 12 weeks with a more pronounced effect in the treatment group. In conclusion, resistance training in combination with supplementation of SCP induced a significantly higher increase in fat-free mass and hand-grip strength than resistance training and placebo supplementation. In addition, there was a significantly higher loss in fat mass and a more pronounced increase in leg strength in the treatment group compared to the control group.

## 1. Introduction

Skeletal muscle mass accounts for approx. 35% of total body weight and plays an essential role in glucose metabolism, endocrine function, thermogenesis, and locomotion [1,2,3,4]. Particularly with regard to the demographic change in western societies, the preservation of muscle mass is of vital importance. An increase in age is well-reported to be associated with a decrease in muscle mass and function [5], which again has a major impact on risk of falls [6] and activity of daily living and thus quality of life [7]. Several studies have also indicated that the loss of muscle mass increases the risk of chronic degenerative diseases of the metabolism (such as type II diabetes mellitus) as well as the orthopedic-accident surgical circuit [8,9,10]. This multi-factorial phenomenon, called sarcopenia [7], has been shown to be prevalent in approximately 25–50% of adults over the age of 60 years [11,12].

Therefore, strategies that can be implemented into primary prevention of this age-induced decline in muscle mass and function need to be identified. Cruz-Jentoft and colleagues (2010) recommend nutritional and exercise interventions as the cornerstones of the prevention and treatment of sarcopenia. In terms of resistance training, it is well established that high loads are necessary to induce beneficial changes in muscle mass and strength [13]. By activating the skeletal muscle protein synthesis, resistance exercise improves muscle protein balance [14]. The mammalian target of rapamycin (mTOR) is generally considered an important regulator of protein synthesis and cell growth [15]. Evidence from several studies suggests that a dietary protein supplementation is an effective strategy for improving strength gains and muscle mass during prolonged resistance exercise in younger and older people [16,17,18]. 

To date, a large number of protein sources including whey [19] or soy protein [20] have been investigated. In this regard, also collagen peptides have gained increasing attention. Several studies have shown that di- and tripeptides such as hydroxyprolyl-glycine (Hyp-Gly) seem to have signaling properties and have shown to induce myoblast differentiation and myotube hypertrophy by activating the mTOR signaling pathway [21]. Hyp-Gly has been found in human blood in considerable amounts following ingestion of collagen peptides [22]. Additionally, the nonessential amino acid glycine, which is also included in collagen peptides, has been reported to have strong anti-inflammatory potential and attenuate muscle wasting in several wasting models (e.g., cancer cachexia or sepsis) [23,24].

In humans, the daily intake of 15 g specific collagen peptides (SCP) has been shown to enhance the adaptive response of a 12-week resistance training program in older men [25]. Indeed, the majority of studies in the field of protein supplementation and RT were conducted with men and there is a lack of studies investigating these effects in women [26]. Therefore, the main objective of this study was to examine the influence of specific collagen peptides on body composition in women following a 12-week RT program. As a secondary aim, isometric strength of the upper and lower extremities, was evaluated. 

## 2. Materials and Methods 

### 2.1. Subjects

In total, 90 premenopausal women aged between 18 and 50 years with a BMI between 20 and 35 kg m^−2^ were randomly assigned to the study groups. We included untrained individuals with no resistance training (RT) experience or who have not been training for several years. Subjects were not eligible if they had any contraindications with regard to physical activity according to American College of Sports Medicine (ACSM) guidelines such as cardiovascular, metabolic, or renal diseases [27]. Inclusion criteria, including premenopausal status, were examined by a comprehensive anamnesis. In addition, the physical constitution of the participants was checked by a detailed physical examination by an experienced sports physician. Moreover, blood was drawn to investigate safety variables such as hemogram, alanine aminotransferase, aspartate transaminase, creatinine, urea, etc. Additionally, participants’ regular physical activity level should not exceed 60 minutes per week assessed with a validated questionnaire [28]. Furthermore, intolerance or allergy against collagen peptides and silicea was an exclusion criterion. Subjects were also excluded from the trial if any health problems during or after physical activity or unstable weight and eating behaviors were present. 

Block randomization was performed using a random number generator [29]. Blinding of investigators and participants was not reversed until all the data had been entered and checked and the statistical analysis had been performed.

**Figure 1:**
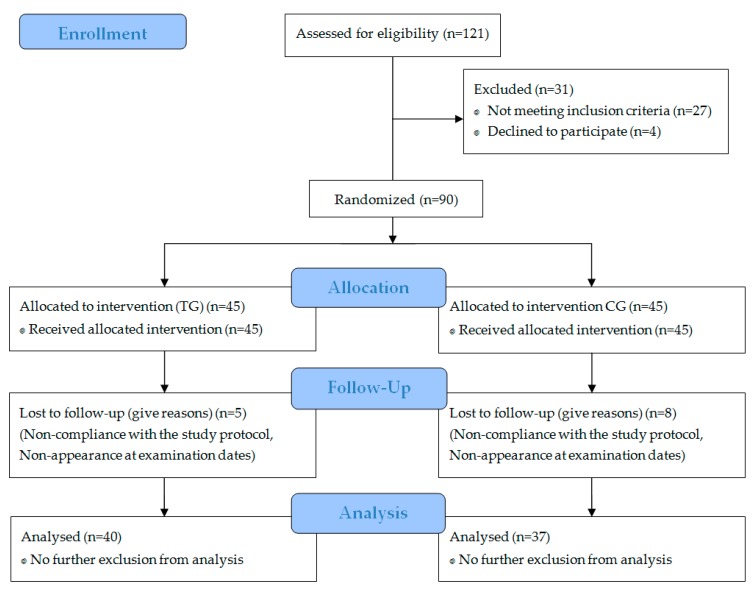
Flow diagram of subject’s recruitment, randomization, and follow up.

Specific data regarding participant recruitment, allocation, and follow-up are presented in Figure 1.

### 2.2. Study Design

The study was designed as a monocentric, prospective, placebo-controlled, double-blinded trial with pre- and post-test measurements conducted at the University of Freiburg, Germany. At a preliminary screening, the inclusion and exclusion criteria were checked using the anamnestic and anthropometric data as well as the results from blood testing. The main outcome of this trial was the differences in fat-free mass (FFM) before and after the resistance training between the treatment group (TG) receiving specific collagen peptides versus the control group (CG). As a secondary outcome, the change in fat mass (FM) was compared between the two groups. Body composition was quantified by using a bioelectric impedance analysis (BIA).

Moreover, maximal isometric strength of the legs and hands were analyzed before and after the 12 weeks. Compliance was checked by collection of returned unused samples. Furthermore, subjects had to document the time of intake of the investigational products in a compliance calendar. The study protocol was approved by the ethical committee of the University of Freiburg. All subjects gave written informed consent. The trial was registered with DRKS-ID: DRKS00013328.

### 2.3. Body Composition

BIA measurements were conducted at first and last visit according to the guidelines of the European Society for Clinical Nutrition and Metabolism (ESPEN) [30]. Examinations were carried out using a seca Medical Body Composition Analyzer 515 (seca© mBCA 515, Hamburg, Germany). The multifrequency device uses four pairs of electrodes that are positioned at each hand and foot. Through the eight-electrode technique, segmental impedance measurements regarding FM and FFM can be estimated [31,32,33]. 

All participants were tested between 7:00 and 11:00 a.m. and adhered to the ESPEN guidelines regarding food and fluid intake in order to enhance reliability and accuracy of the measurements [30]. Before the body composition analyses, body height was obtained with the Stadiometer (seca© 274 Hamburg, Germany) to the nearest mm with an accuracy of ±5 mm. Impedance was measured with a current of 100mA at a frequency of 50 kHz. Details of the BIA protocol are described in the validation study from Bosy-Westphal and colleagues (2013) [31].

### 2.4. Maximal Isometric Strength

Changes of maximal isometric leg strength were conducted via a 90° leg press device with an integrated foot force platform (Kistler®, Winterthur, Schweiz) constructed by the University of Freiburg, Germany. Thereby, subjects were in the supine position with a 90° angle in the hip, knee, and ankle. Before actual testing, subjects were instructed to complete four sets with submaximal load in order to ensure a proper warm-up and familiarization. Subsequently three actual maximal isometric leg strength tests were performed. Subjects were asked in every attempt to realize their maximum power as quickly as possible. Single attempts were separated by a 4-min resting period to ensure adequate recovery. The best of the three measurements was used in statistical analyses [34]. 

Maximal isometric grip strength was assessed with a hand dynamometer (TL-LSC100, liteXpress GmbH, Coesfeld, Germany) according to the recommendations of the Southampton protocol [35,36].

### 2.5. Physical Activity

Subjects were advised not to change their regular activities apart from the prescribed and supervised resistance training sessions (3 day/week with at least one regeneration day in between sessions). Resistance training took place at the University of Freiburg and was supervised by experienced physical exercise instructors. For 12 weeks, the participants trained for 60 minutes three times per week. After a 10 min warm-up on a cycle ergometer (50–100 W), the participants completed a three-set full-body workout (leg press, back trainer, lat pull-down exercise, sit-up, and chest press) with the following number of repetitions: week 1–2: 15, week 3–4: 12, week 5–8: 10, week 9–12: 8. Adapted to the proper execution of the required repetitions the intensity was adjusted individually. Missing more than six training sessions resulted in exclusion from further participation.

### 2.6. Nutrition

The subjects were instructed not to alter their dietary habits during the intervention. Nutritional status was examined using Nutriguide 4.6 (Nutri Science GmbH Hausach, Germany). With the aid of an online platform, subjects protocolled their dietary behavior over three consecutive days (two week days and one weekend day), both before and after the intervention. 

Additionally, randomly assigned, the subjects received 15 grams of specific bioactive collagen peptides (Bodybalance®, Gelita AG, Eberbach, Germany) or a placebo (silicon dioxide) similar in taste. The participants were instructed to dissolve the products in 250 mL water and to drink the solution within 60 min after finishing the exercise program or at the same time on non-training days.

### 2.7. Statistical Analysis

The statistical evaluation based on the data from all participants completing the trial and keeping the protocol conditions (per protocol population). 

Software package SPSS version 24.0 (IBM, Armonk, NY, USA) was applied for all statistical analyses. Data are presented as mean (M) ± standard deviation (SD) in tables and figures. Since the variable data of all groups showed normal distribution according to the results of Kolmogorov–Smirnoff (KS)-test, the homogeneity of the baseline values between study groups was checked via independent *t*-tests. Significant interaction was tested by two-way repeated measures ANOVA with time and condition as main factors. In order to describe, if effects have a relevant magnitude, Cohen’s d was calculated. Potential covariates were examined using descriptive statistics at baseline. If differences between the experimental groups were found at baseline, these factors were included as covariates in an ANCOVA. Changes in body composition and muscle strength during the intervention period within groups were analyzed with help of paired samples *t*-test. The significance level was set to α = 0.05 for all performed two-sided tests. 

## 3. Results

### 3.1. Subject Characteristics

A total of 77 subjects completed the investigation and were included in the per protocol analysis. Neither the TG nor the CG differed significantly in age, height, weight, or body mass index (BMI) (Table 1). All 13 dropouts failed to comply with the study design, mainly missing too many training days. No dropouts occurred through side effects of the supplemented SCP or placebo. In addition, no pathological findings were observed in the routine blood test.

### 3.2. Body Composition

**Figure 2:**
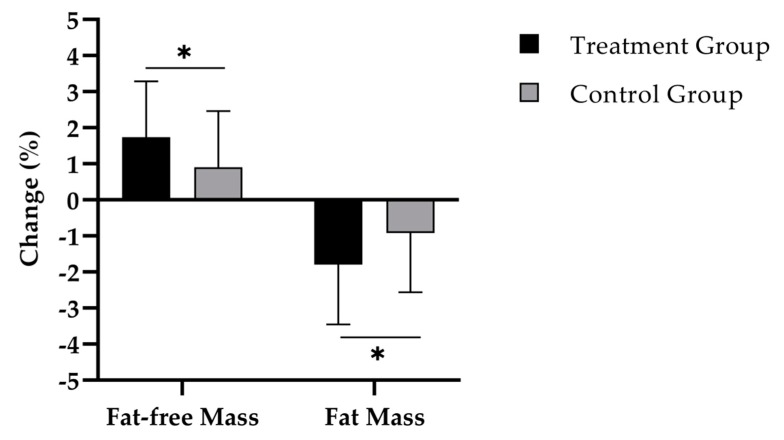
Change in percentage fat-free mass and fat mass after 12 weeks of intervention in treatment group (*n* = 40) and control group (*n* = 37). Repeated measures analysis of variance (RMANOVA). * = *p* < 0.05. Data are expressed as mean (M) and standard deviation (SD).

Figure 2 shows that the TG had a statistically significantly higher increase of percentage fat-free mass compared to the CG (1.8 ± 1.6% vs. 0.9 ± 1.6%; d = 0.55) (Table 2). This percentage change equals an absolute gain of 1.0 ± 0.9 kg in the TG and 0.4 ± 0.9 kg in the CG. 

In addition, a significant decline of percentage body fat was observed in both TG (*p* < 0.001) and CG (*p* < 0.01), with a statistcally significantly higher fat reduction in TG (*p* < 0.05; d = 0.54) (Table 2). Translating these findings in absolute values, the TG group (−1.5 ± 1.7 kg) also showed a higher reduction of absolute FM compared to the CG (−0.7 ± 1.6 kg).

The results and the statistical analysis are based on a per protocol (PP) analysis. This type of analysis was chosen since we believe that the effects of an intense 12 weeks exercise regimen are better reflected in those subjects who participated in this program on a regular basis. In addition, no side effects were reported regarding the supplements and none of the 13 dropouts was related to supplement intake. Nevertheless, we also performed an intention to treat (ITT) analysis including all 90 women applying a multiple inputation approach. In this analysis, we could also show comparable results with a significant difference in the RMANOVA analysis regarding FFM (∆ TG vs. CG: +0.8 %; *p* < 0.05), FM (∆ TG vs. CG: −0.9 %; *p* < 0.05) and hand grip strength (∆ TG vs. CG: +1 kg; *p* < 0.05).

### 3.3. Muscle Strength

**Figure 3:**
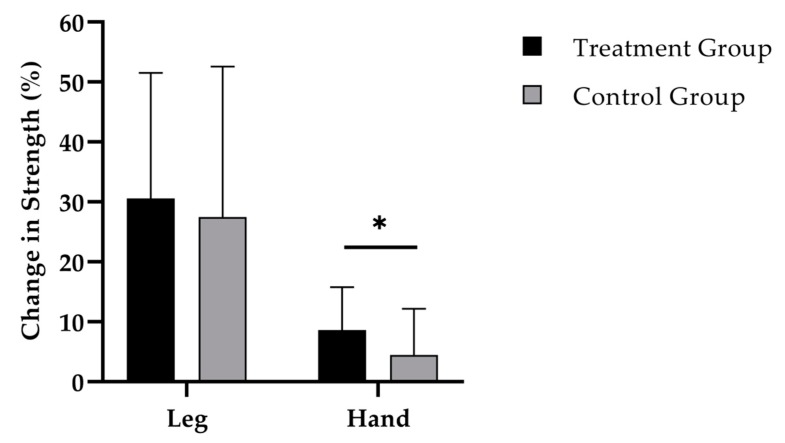
Change in leg and hand-grip strength after 12 weeks of intervention in treatment group (*n* = 40) and control group (*n* = 37). Repeated measures analysis of variance (RMANOVA). * = *p* < 0.05. Data are expressed as mean (M) and standard deviation (SD).

While the 12-weeks administration of SCP (Figure 3) was accompanied by an increase in leg strength to 250.8 (SD 147.3) N (*p* < 0.001), a raise of 233.5 (SD 182.9) N was shown in the CG (*p* < 0.001). No statistically relevant difference was observed between the groups. 

Finally, hand-grip strength increased by 2.7 (SD 2.1) kg (*p* < 0.001) in the TG (Figure 3). In the training group without SCP supplementation an increase by 1.3 (SD 2.4) kg (*p* < 0.01) was observed. Change in hand-grip strength was significantly different between the groups (*p* < 0.05; d = 0.63).

### 3.4. Dietary Intake

The analysis of dietary behavior was only performed for macronutrient and energy intake. Baseline data showed a significant difference between the groups for carbohydrates and fats (*p* < 0.05).

No significant differences for dietary intake of macronutrients and energy were found before and after the intervention in the TG. Mean changes for energy, carbohydrate, fat, and protein intake were −91.8 ± 470.0 kcal, −2.7 ± 57.9 g, −7.9 ± 31.6 g, and −1.9 ± 21.2 g, respectively. The analysis of the nutritional protocols for the CG revealed no significant changes for energy 4.1 ± 433.8 kcal and protein intake 0.5 ± 23.2 g. However, there was a significant change for carbohydrate −19.4 ± 53.6 g (*p* < 0.05) and fat intake 7.3 ± 23.9 g (*p* < 0.05) within the group.

## 4. Discussion

The main outcome of the present study was that supplementation with specific collagen peptides significantly enhanced the positive effects of resistance training in premenopausal women regarding the increase in fat-free mass and hand-grip strength and a decrease in fat mass. 

Meta-analyses from controlled clinical trials predominantly in men have suggested a synergistic effect of resistance training and protein supplementation [16,37,38]. Interestingly, studies investigating the effect of protein supplementation and resistance exercise in women, and in particular on non-athletic premenopausal women, are scarce [26]. Most studies in women have been performed with postmenopausal women, some of them investigating the effects of different exercise regimens, others studying the influence of different nutrients including protein [39,40]. Previous investigations found improvements in metabolic parameters, muscle strength, or body composition following RT and protein supplementation. However, the effects on fat or muscle mass were rather small. In a current systematic review and meta-analysis, it was revealed that the effect of whey protein supplementation on fat free mass in young and old women in combination with a resistance training were +0.23 kg [26]. However, the calculation included both female athletes and postmenopausal women. A target group comparable with the present investigation was missing. In addition, results from postmenopausal women should not be compared to respective effects in premenopausal women. After menopause, the drop in estrogen levels but also the decrease in testosterone levels with ageing impairs hypertrophic muscular adaptations in postmenopausal women [41,42]. 

Before menopause, although systematic gender-based comparisons are missing, it is generally believed that protein metabolism and anabolic muscular response following RT are not significantly different between men and women [43]. Nevertheless, since women have a lower total muscle mass, the exercise-induced increase in muscle mass and strength are generally smaller in women. In a recent meta-analysis, the additional effect of protein supplementation in men has been designated by +0.3 kg [44]. With regard to these data, the results of the present study indicated not only a similar trend, but larger increases in FFM.

In the past, the majority of investigations have been performed with proteins such as whey, casein, soy protein, or isolated amino acids. Recently, improvements in body composition have been described in elderly men following a 12-weeks resistance training combined with the supplementation of 15 g collagen peptides [25]. The results of the present investigations in principle confirm previous results, although the magnitude of respective effects was smaller, as expected, taking gender-related difference into account. 

There is an ongoing debate regarding the physiological trigger for muscular hypertrophy following resistance exercise and SCP supplementation, since the amount of some essential amino acids and in particular leucine is rather low in collagen peptides. Previous studies have shown that changes in muscle mass induced by resistance training were associated with the mTOR signaling pathway. Several investigations have demonstrated that predominantly branched chained amino acids (BCAA) such as leucine, trigger the activation of the mTOR signaling pathway [45,46]. However, it has been shown that the mTOR complex is also activated by arginine and glycine. Collagen peptides show a high arginine and especially glycine content, therefore, also these amino acids in SCP might stimulate mTOR signaling, as recently demonstrated in cell culture experiments [47,48]. 

Moreover, collagen peptides are absorbed in the small intestine to a considerable amount in peptide form [49,50,51]. These bioactive peptides act as signaling molecules that trigger anabolic processes in several tissues and, in particular, in skeletal muscle [25,52].

It has recently been shown that hydroxyprolyl–glycine (Hyp-Gly)-peptides have strong signaling properties and have shown to induce myoblast differentiation as well as myotube hypertrophy by activating the mTOR signaling pathway [21]. 

Besides mTOR stimulation, it could be speculated that part of the observed increase in FFM in this study could be related to an increase in the collagen content of the intramuscular connective tissue. Approximately 10% of the skeletal muscles consist of collagen, which is a major contributor to the function and the biochemical structure of skeletal muscles [53,54]. Various studies have demonstrated that the synthesis of collagen and other extracellular macromolecules is stimulated by specific collagen peptides [49,50,55,56]. 

With regard to the alterations in percentage fat mass (FM), the specific collagen peptides group showed a statistically significant higher decrease compared to the control group. The divergence of FM in this trial might perhaps be partly explained by a higher resting energy expenditure caused by the more pronounced gain in FFM in the group following specific collagen peptides administration. 

Although there is currently limited knowledge on the effects of collagen peptides on FM, preclinical trials have confirmed that a supplementation with collagen peptides reduces adipocyte size [57].

Nevertheless, the study has some limitations. Although Dual X-ray absorptiometry (DXA) is a commonly used method for the assessment of body composition [58], the present investigation estimated FM and FFM by using BIA (Seca© mBCA 515, Hamburg, Germany). This was partly attributable to the fact that the application for DXA presents a radiation hazard to healthy participants, which is not in accordance with national ethical guidelines. On the other hand, recent investigations demonstrated that BIA equations based on magnetic resonance imaging were an accurate tool to estimate body composition in healthy adults, which can be compared with the validity, reproducibility, and precision of other two-compartment reference methods, such as DXA [31,32,33]. 

While data from the nutritional protocols revealed that dietary behavior did not change significantly in the TG, baseline data between the groups as well as carbohydrate and fat intake before and after the intervention were significantly different in the CG. However, the physiological relevance of these differences is likely to be small. 

In future studies, the mode of action of how collagen peptides stimulate muscle anabolism and their impact on muscular and fat metabolism needs to be further elucidated. The amount and composition of single amino acids is not likely to fully explain the effects found in this study. The next step would be to focus on the role of collagen peptides in the direct regulation of anabolic or catabolic processes on a cellular level by muscle biopsy studies. Possible pathways that should be investigated include the stimulation of collagen synthesis, the mTOR and AMP-activated protein kinase (AMPK) pathway, or downregulation of pro-inflammatory processes.

## 5. Conclusions

In conclusion, the resistance training regimen in combination with supplementation of specific collagen peptides induced a significantly higher increase in percentage FFM and a decrease in percentage FM compared to placebo. The positive impact on body composition also resulted in an improved strength, as indicated by a significant increase in hand-grip strength compared to the group without collagen peptide supplementation.

## Figures and Tables

**Table 1:** Descriptive and anthropometric characteristics of the subjects in TG (*n* = 40) and CG (*n* = 37).

Parameter	Group	(M ± SD)	MIN	MAX
Age (years)	TG	38.3 ± 8.7	18.0	50.0
	CG	41.6 ± 6.9	25.0	49.0
Height (cm)	TG	167.0 ± 6.1	154.0	183.0
	CG	165.8 ± 5.6	150.0	179.0
Body weight (kg)	TG	73.5 ± 10.7	59.6	94.2
	CG	72.8 ± 10.1	58.9	103.5
BMI (kg m^−2^)	TG	26.4 ± 3.8	21.0	35.0
	CG	26.5 ± 3.4	21.9	34.5

Data are expressed as mean (M) and standard deviation (SD). TG = treatment group; CG = control group.

**Table 2:** Body composition and muscle strength in the subjects before (pre) and after (post) the intervention in the treatment group (TG) or control group (CG).

Parameter	Group	Pre	Post	*p*-Value ^1^	RMANOVA ^2^
Body weight (kg)	TG	73.5 ± 10.7	73.0 ± 10.4	NS	NS
	CG	72.8 ± 10.1	72.4 ± 10.4	NS	
Fat-free mass (%)	TG	62.6 ± 6.0	64.4 ± 6.2	<0.001	<0.05
	CG	63.8 ± 6.0	64.7 ± 6.0	<0.01	
Fat mass (%)	TG	37.4 ± 6.0	35.6 ± 6.2	<0.001	<0.05
	CG	36.2 ± 6.0	35.3 ± 6.1	<0.01	
Leg strength (N)	TG	890.2 ± 246.4	1141.0 ± 288.8	<0.001	NS
	CG	939.8 ± 312.0	1173.3 ± 361.9	<0.001	
Hand-grip strength (kg)	TG	31.7 ± 3.5	34.4 ± 3.8	<0.001	<0.05
	CG	32.7 ± 6.0	34.0 ± 5.5	<0.01	

^1^ Paired *t*-test. ^2^ Repeated measures analysis of variance (RMANOVA) two-way. Data are expressed as mean (M) and standard deviation (SD). NS = not significant.
